# Single-Nucleotide Polymorphisms in MICA and MICB Genes Could Play a Role in the Outcome in AML Patients after HSCT

**DOI:** 10.3390/jcm10204636

**Published:** 2021-10-09

**Authors:** Alena Machuldova, Lucie Houdova, Katerina Kratochvilova, Martin Leba, Pavel Jindra, Pavel Ostasov, Diana Maceckova, Robin Klieber, Hana Gmucova, Jiri Sramek, Monika Holubova

**Affiliations:** 1 Laboratory of Tumor Biology and Immunotherapy, Biomedical Center, Faculty of Medicine in Pilsen, Charles University, 323 00 Pilsen, Czech Republic; pavel.ostasov@lfp.cuni.cz (P.O.); maceckod@lfp.cuni.cz (D.M.); KLIEBERR@lfp.cuni.cz (R.K.); 2 Department of Histology and Embryology, Faculty of Medicine in Pilsen, Charles University, 301 66 Pilsen, Czech Republic; SRAMEKJ@fnplzen.cz; 3 NTIS, Faculty of Applied Sciences, University of West Bohemia, 301 00 Pilsen, Czech Republic; houdina@ntis.zcu.cz (L.H.); kkratoch@ntis.zcu.cz (K.K.); lebam@kky.zcu.cz (M.L.); 4 Department of Haematology and Oncology, University Hospital Pilsen, 304 60 Pilsen, Czech Republic; jindra@fnplzen.cz (P.J.); GMUCOVAH@fnplzen.cz (H.G.)

**Keywords:** allogeneic hematopoietic cell transplantation, graft-versus-host disease, graft-versus-tumor effect, NKG2D, MICA, MICB, NK cells

## Abstract

NKG2D and its ligands, MICA and MICB, are known as the key regulators of NK cells. NK cells are the first reconstituted cells after the allogeneic hematopoietic stem cell transplantation (HSCT); therefore, it is crucial to understand their role in HSCT outcome. In the presented study, we investigated the single amino acid changes across the exons 2–4 of MICA and MICB genes, and point mutations within the NKG2D gene, which defines the type of NKG2D haploblock (HNK/LNK) in the donors (*n* = 124), as well as in patients with acute myeloid leukemia (*n* = 78). In our cohort, we found that graft from a donor with at least one MICA allele containing glycine at position 14 (MICA-14Gly) is significantly associated with deterioration of a patient’s overall survival (OS) (*p* < 0.05). We also observed a negative effect of MICB-58 (Lys → Glu) polymorphism on relapse-free survival (RFS), although it was not statistically significant in multivariate analysis (*p* = 0.069). To our knowledge, this is the first work describing the role of MICA-14 and MICB-58 polymorphisms on HSCT outcome.

## 1. Introduction

Allogeneic hematopoietic stem cell transplantation (HSCT) is the only curative treatment for many patients with myeloid malignancies, mostly in patients with acute myeloid leukemia (AML). Despite the significant progress in post-transplant therapy, HSCT is still accompanied by multiple complications. Particularly, graft-versus-host disease (GVHD), infections, and relapse of the disease are the most common and serious [1].

The recovery of the immune system is critical for the success of HSCT. Natural killer (NK) cells are the first lymphocytes reaching the standard numbers within the first few weeks post-transplantation [2], and, therefore, they play a key role in hematopoiesis during the first months after HSCT. NK cells are recognized as crucial cells for early relapse control, and recent data have demonstrated that NK cells also affect GVHD development [3,4,5]. The exact role of NK cells and crucial parameters, which could help us to improve the outcome of HSCT, are currently broadly investigated. 

NK cells’ activity is driven via inhibitory and activating receptors [6]. The best-known regulatory receptors of NK cells are the killer immunoglobulin-like receptors (KIR) interacting with their ligands—HLA molecules [7]. Because the virus-infected cells or tumor cells downregulate HLA molecules, KIR cannot interact with inhibitory receptors, and NK cells are activated more easily through multiple activating receptors, e.g., NKG2D [7]. NKG2D binds stress-induced ligands (MICA, MICB, and ULBP/RAET) present predominantly on damaged cells [8]. NKG2D is a C-type lectin receptor present on NK cells and subsets of T cells [9]. Its gene was described as strongly evolutionarily conserved, but four haplotype alleles playing a role in cytotoxicity have been described—LNK1, LNK2, HNK1, and HNK2. Whether the allele is LNK1 or HNK1 is determined by rs1049174 polymorphism (C → G), while LNK2 vs. HNK2 is determined by polymorphism rs2255336 (G → A). LNK1 and LNK2 initiate low NK cytotoxicity, while HNK1 and HNK2 trigger high NK cytotoxicity. Six different allele combinations create two haploblocks—LNK1/LNK1, LNK1/HNK1, and HNK1/HNK1 form haploblock 1 (Hb-1); LNK2/LNK2, LNK2/HNK2, and HNK2/HNK2 form haploblock 2 (Hb-2) [10]. The haplotype HNK1/HNK1 is associated with a decreased risk of cancer development compared to the LNK1/LNK1 [10]. From the HSCT perspective, transplantation with HNK1/HNK1 donors is related to lower transplantation-related mortality and better OS [11].

The predominant NKG2D ligands, MICA and MICB, are highly polymorphic genes with the main variability in the exons 2–4 encoding extracellular (receptor-binding) domains α1, α2, and α3 [12]. The best-known functional polymorphisms with a role in HSCT are in exon 3 of MICA and MICB, called MICA-129 and MICB-98 [13]. MICA-129 with methionine binds NKG2D receptor with higher affinity than the variant with valine causing 1) faster and more intensive activation of NK cells associated with a higher risk of acute GvHD, and 2) the coherent early exhaustion of the receptor increasing the risk of relapse [14]. On the other hand, carriers of MICA-129 with valine are at a higher risk of chronic GVHD because of the longer activity of NK cells [15]. A mismatch between donor and patient in MICA-129 amino acid was described to increase the risk of acute GVHD development, as well as for MICB-98 [16,17,18]. Accordingly, a lower risk of acute and chronic GVHD was described when full-MICA-allele was matched [17,19]. These results indicate an important role of MICA/MICB gene polymorphisms in HSCT, which should be further studied. 

The present study investigates the impact of polymorphisms in the NKG2D gene and the most important exons of its ligands MICA and MICB on the clinical outcome of patients undergoing HSCT.

## 2. Materials and Methods

### 2.1. Cohort Description

Clinical data (detailed in Table 1) were collected from 124 patients with myeloid malignancies after HSCT and their donors at the Department of Haematology and Oncology, University Hospital Pilsen. The samples corresponding donors’ cells (*n* = 124) were collected from peripheral blood of patients after the HSCT when donor hematopoiesis was fully regenerated (confirmed by determination of the 100% level of the chimerism using the variable number of tandem repeats (VNTR) estimation by fragment analysis during a routine examination). In a part of the transplanted patients (*n* = 78), pretransplant samples (corresponding to the patients’ DNA) were obtained as well.

### 2.2. Sample Preparation and Sequencing

DNA was extracted with the Maxwell 16 Blood DNA Purification Kit (Promega, WI, USA) according to the manufacturer´s protocol. PCR cycles were set up to denaturation for 94 °C for 1 min, with 35 cycles consisting of denaturation (94 °C for 15 s), primer annealing (for NKG2D 58.5 °C for 15 s and MICA and MICB 61 °C for 15 s), and extension (in the case of NKG2D, 72 °C for 60 s, and MICA and MICB 72 °C for 120 s). The last extension lasted 10 min at 72 °C for all samples. Total volume of the reaction was 25 µL of the following: 12.5 µL 2 × LA Hot Start Master Mix (Top-Bio, Vestec, Czech Republic), 1 µL forward primer, 1 µL reverse primer, 8.5 µL water, and 2 µL DNA. Primers and corresponding fragment lengths are shown in Table 2.

### 2.3. Sequence Evaluation

We were looking for the point mutations of NKG2D which led to a different NK cell activity, while, for MICA/MICB, we used complete sequences of exons 2–4 of both genes for the next analyses.

The point mutation in NKG2D was evaluated within the exon 8 of NKG2D at position 10,372,766 of chromosome 12 (rs1049174) and within the exon 4 of NKG2D at position 10,379,727 of chromosome 12 (rs2255336) [25]. Therefore, only the corresponding parts of the NKG2D gene were sequenced.

In the case of the exons 2–4 of MICA and MICB genes, the reverse and forward sequences of the individual samples were combined in a CAP3 tool [26] and aligned by the Clustal Omega tool within the UGENE program [27] to reference sequences downloaded from the IPD database (3.42 version) [28]. The sequences were then cut into the individual exons by a custom script in Python. The resulting sequences containing ambiguous nucleotides were considered heterozygous. These sequences were then deconvoluted into the combination of the two most common reference sequences. This was achieved by duplication of sequences, with both nucleotide options at each ambiguous position. This was repeated until all ambiguous positions were resolved. Subsequently, all possible sequences were compared with reference sequences, and those without corresponding reference sequences were removed. From the remaining ones, the pairs whose combination would recreate original ambiguous sequences were selected. Sequences that did not meet any of the above conditions were analyzed individually.

The set of all sequences (the sample sequences and the reference sequences downloaded from the IPD database) was then translated into the amino acids by using the “translate” tool at bioinformatics.org [29] for all individual exons. The correctness of the translation was controlled by comparing the results of translation with the amino acid sequence of MICA (sequence Q29983) and MICB (Q29980) available at UniProt [30]. All the following analyses were done with amino acid sequences. The first step with the amino acid sequences of each exon was to cluster them into groups. The groups were created according to the sequence differences by using reference sequences. Each sequence from our dataset was then included in the group with the same sequence. The second step was to label the sequences of the individuals according to the group the sequences fall into. Samples belonging to one group (homozygous within a particular exon) were labeled as Sx/Sx (e.g., S1/S1); samples with two different sequences of an exon detected were labeled as Sx/Sy (e.g., S1/S3). In our analyses, we labeled individuals as fully homozygous only in the case that the sample was homozygous across all three exons.

The combinations mentioned in Table 3 below were then tested by the Kaplan–Meier method for overall survival and relapse-free survival.

### 2.4. Statistical Analysis

Only the combinations of groups containing at least 5 samples were used for multiple-combinations statistical analyses (for example, S1/S1 vs. S1/S2, S1/S4, and S1/S6). The role of presence/absence of a particular group (e.g., samples containing S1 versus samples without the presence of S1) was evaluated in all enrolled samples.

Analyses of the effect of the groups on OS and RFS, and visualizations were performed using Kaplan–Meier analysis and log-rank testing initially. The statistical analysis was performed using R (version 4, The R Foundation for Statistical Computing, Vienna, Austria) [31] and packages survival (version 3.2) and survminer (0.4.8). Univariate Cox regression was used for the assessment of the effect of clinical parameters on OS and RFS. Statistically significant clinical parameters from univariate analysis were then used for multivariate Cox regression (DRI, AML as secondary malignancy, karyotype, disease status during HSCT, and cGVHD for OS, as well as for RFS). Results with a *p*-value lower than 0.05 were assessed as statistically significant.

## 3. Results

### 3.1. Clinical Data Evaluation

The demographics of the study population are shown in Table 1 in Section 2. The median post-transplant follow-up was 16 months (range 0.5–143 months). The OS was 63% at year 1, 27% at year 3, and 14% at year 5. RFS after 1, 3, and 5 years was 56.5%, 27%, and 14.5%, respectively. The cumulative incidence of non-relapse mortality (NRM) was 12% at 1 year, and 20% at 5 years. The cumulative incidence of relapse was 17% and 27% at years 1 and 5, respectively. The most common causes of death were relapse (48% of all deaths) and infection (26%), followed by organ failure (11%), GVHD (10%), and one patient died due to graft rejection.

The typical correlation between the clinical parameters and patients´ outcomes was observed. A relationship between disease risk score (disease risk index, DRI, [20]) and RFS and OS was observed (both *p* < 0.0001). Contrastingly, the EBMT risk score [32] did not predict longer or shorter RFS or OS (RFS *p* = 0.62 and OS *p* = 0.23). Shorter RFS was highly associated with the presence of complex karyotype (81% of all patients with complex karyotype had relapse and 69% of all patients with complex karyotype died due to relapse). The most common cause of death in patients with normal karyotype was infection (43%), followed by relapse (29%). Another negative factor in our cohort was previous malignancy, causing poorer OS (*p* < 0.005) with worse relapse-free survival (*p* < 0.05). The same was observed in patients with active disease present at the time of the HSCT, compared to patients in complete remission (OS *p* < 0.05, RFS *p* < 0.05). Patients with acute GVHD (aGVHD) grade III–IV (*n* = 13) have significantly shorter OS than patients with aGVHD grade I–II (*n* = 15) (*p* < 0.01). Oppositely, having chronic GVHD (cGVHD) seems to be protective in comparison with no cGVHD from an OS perspective (*p* < 0.05). Univariate analysis was carried out for all relevant clinical parameters, and results of this analysis are shown in Table 4.

### 3.2. Distribution of Exon Groups and Polymorphisms within the Cohort

The amino acid sequences were distributed into the groups based on the sequences’ similarity (for details see Materials and Methods). The amino acid reference sequences of exon 2 of MICA create 10 groups (for amino acid reference sequences´ distribution see Supplementary Tables S1–S6). The amino acid sequences of donors and patients belonged to the groups S1, S2, S4, S6, and S7. Exon 3 of MICA was divided into 18 groups; 10 groups were detected within our samples (S1, S2, S5–S9, S13, S15, and S16). The reference amino acid sequences of exon 4 of MICA create 10 groups. The sequences from our dataset fall into the groups S1–S3, S5, S6, and S10. The reference amino acid sequences of exon 2 of MICB created the six groups, as well as for exon 3 of MICB. Exon 4 has three different groups. All amino acid sequences of our samples belong to the groups S1, S2, and S3 for all three exons, except one donor who had S5 within exon 2 of MICB.

In our dataset, 23% of donors and 23% of patients were homozygous within all sequenced exons 2–4 of MICA. The number of homozygous samples on the exon level were similar between patients and donors; 49% of all samples (48% of donors and 50% of patients) were homozygous within exon 2 of MICA, 28% (27% of donors and 28% of patients) within exon 3 of MICA, and 36% (35% of donors and 38% of patients) within exon 4 of MICA.

Regarding the MICB homozygosity, 23% of all donors and 28% of all patients were homozygous in MICB. On the exon level, 39% of all samples (34% of donors and 47% of patients) were homozygous within exon 2 of MICB, 49% (49% of donors and 49% of patients) within exon 3 of MICB, and 87% (88% of donors and 85% of patients) within exon 4 of MICB.

The rest of the samples were heterozygous with different combinations (two exons heterozygous and one homozygous, or other combinations) where we cannot say which combination of alleles of donor or patient is correct.

NKG2D Hb-1 and NKG2D Hb-2 were evaluated as well. In our cohort, 9% donors and 10% patients had HNK1/HNK1 combination, 38% donors and 49% patients had LNK1/HNK1, and 53% donors and 41% patients had LNK1/LNK1 haplotype. Regarding the Hb-2, only 3% of donors and 1% of patients carry HNK2/HNK2, 27% of donors and 38% of patients HNK2/LNK2, and the most represented combination was LNK2/LNK2 with 70% of donors and 61% of patients.

### 3.3. Association of Polymorphism with Clinical Data

#### 3.3.1. Donor MICA Exon 2 Plays a Role in Overall Survival

In the donors´ cohort containing 124 samples, we found four homozygous MICA exon 2 combinations (S1/S1, S2/S2, S4/S4, and S6/S6) where S1/S1 was the most frequent one (52 samples). In the heterozygotes group, three groups were mostly detected—S1/S2 (13 samples), S1/S4 (25 samples), and S1/S6 (9 samples). The rest of the groups were too rare (1–5 samples) to be statistically evaluated.

**Figure 1:**
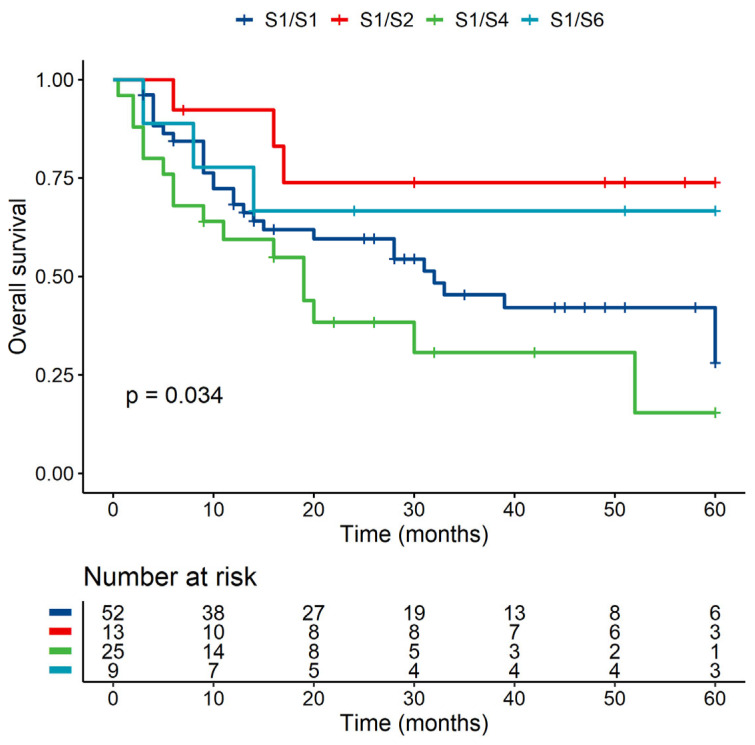
Kaplan–Meier OS analysis of groups within exon 2 of MICA.

Kaplan–Meier OS analysis of S1/S1, S1/S2, S1/S4, and S1/S6 shows a statistically significant difference between individual groups on OS (*p* < 0.05) (Figure 1). The two worst group combinations—S1/S1 and S1/S4—were then examined by multivariate analysis to confirm that no clinical parameter is responsible for this difference. This analysis showed no effect of the clinical parameters and a 2.8 times higher risk of death for patients with graft S1/S4 than with S1/S1 combination (HR = 2.745 (95% CI, 1.113–6.771, *p* < 0.05)).

**Figure 2:**
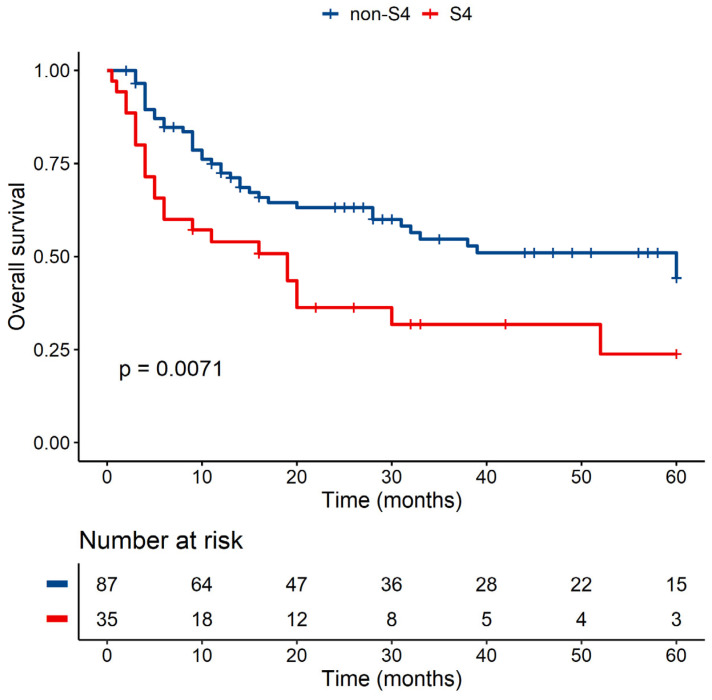
OS of patients transplanted with graft containing at least one copy of MICA-14Gly (S4) versus grafts lacking MICA-14Gly group of MICA exon 2 (non-S4).

From the additional analysis of the presence/absence of a particular group, we confirmed the linkage of group S4 with significantly shorter OS (with *p* < 0.01 for univariate analysis and *p* < 0.05 for multivariate analysis with HR = 2.254 (95% CI, 1.058–4.801)) compared to non-S4 groups (Figure 2). The percentage rate of survivors and dead patients with causes of death are summarized in Table 5. The univariate analysis was carried out for the other groups, but no statistically significant difference was observed with them (*p* = 0.61 for S1 *n* = 104 vs. non-S1 *n* = 17; *p* = 0.1 for S2 *n* = 18 vs. non-S2 *n* = 103; *p* = 0.07 for S6 *n* = 13 vs. non-S6 *n* = 108).

We found that the S4 group has glycine at position 14 of MICA, which distinguishes the S4 group from the others which have tryptophan at this position (Table 6).

#### 3.3.2. Patients’ Homozygosity within MICB Seems to Be Linked to a Lower Risk of Relapse in Univariate but Not in Multivariate Analysis

**Figure 3:**
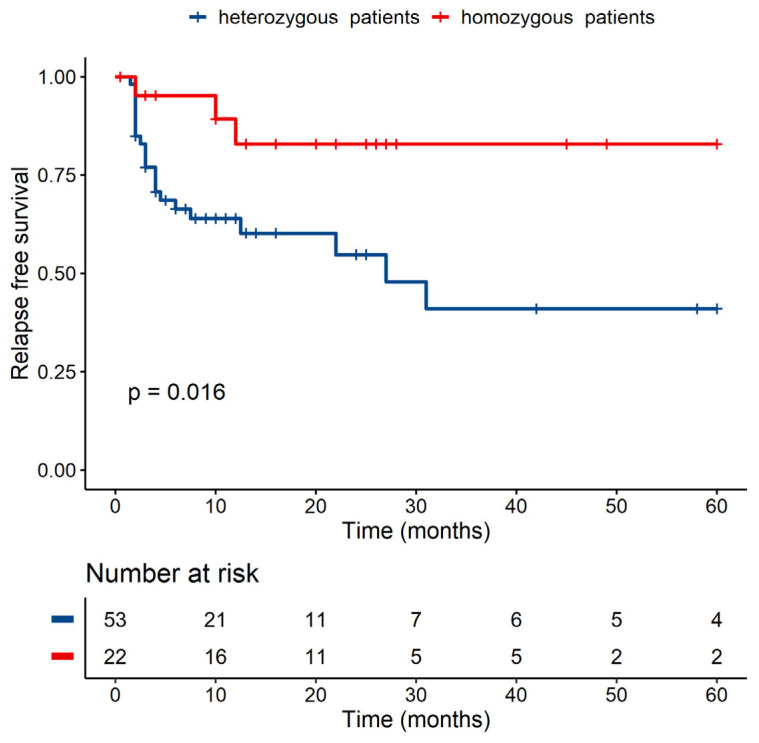
RFS of MICB homozygous versus heterozygous patients using univariate analysis.

**Figure 4:**
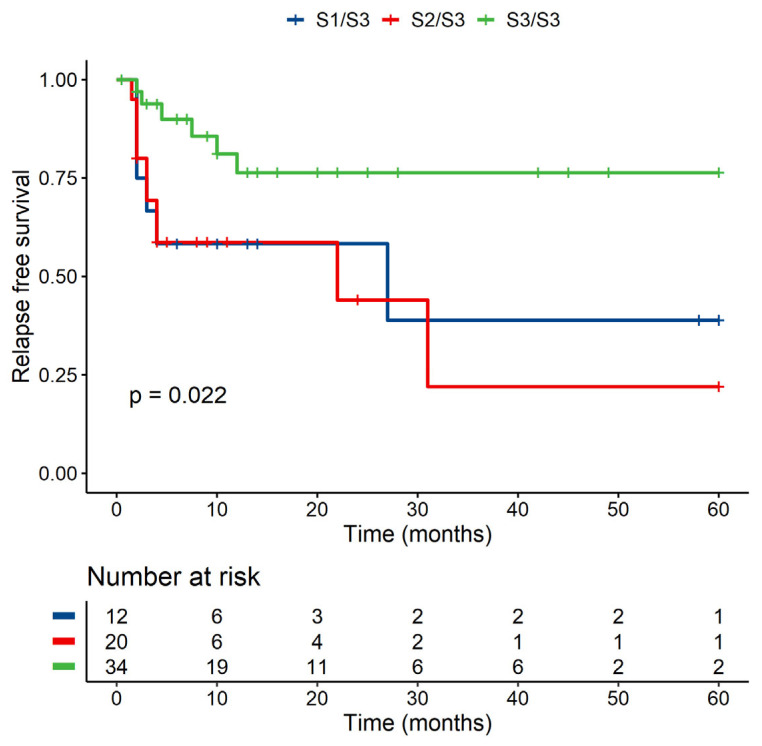
Univariate analysis—RFS of patients according to their exon 3 of MICB.

A statistically significant difference was detected in RFS between MICB homozygous and MICB heterozygous patients (*p* < 0.05) (Figure 3), and a similar situation can be seen in the case of MICB exon 3, where heterozygous patients with two different groups (S1/S3 and S2/S3) have worse RFS than patients with S3/S3 with *p* < 0.05 in univariate analysis (Figure 4). None of these analyses were confirmed by multivariate analysis (for MICB homozygosity vs. heterozygosity, *p* = 0.322, for MICB exon 3 *p* = 0.376). In the donors group, no significant role of homozygosity/heterozygosity for RFS or OS was observed (data not shown).

#### 3.3.3. MICB-58Lys Can Be Linked to a Lower Risk of Relapse

**Figure 5:**
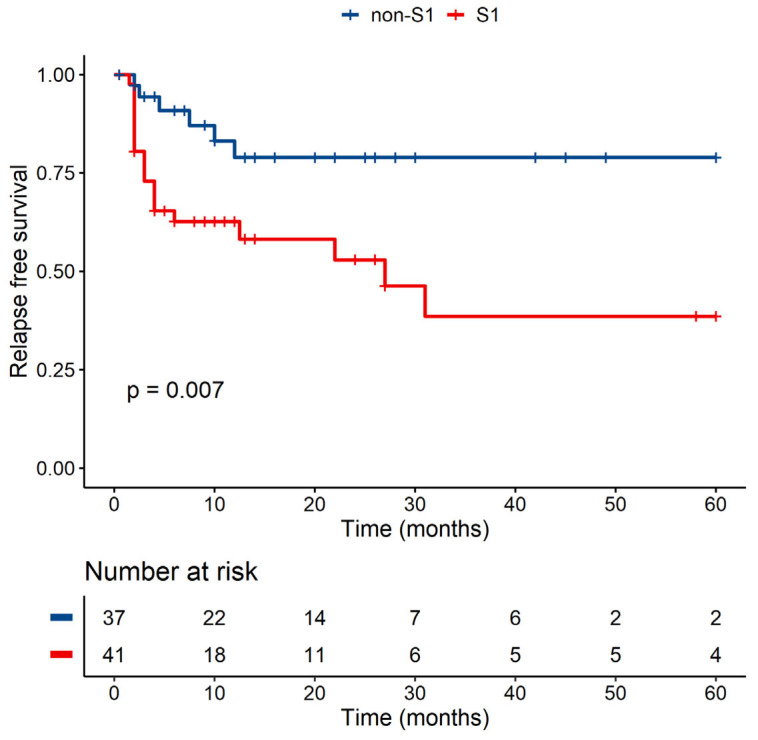
Univariate analysis—RFS of patients with at least one copy of MICB-58Glu (S1) versus patients lacking MICB-58Glu (non-S1) group of MICB exon 2.

We observed the role of the presence/absence of a specific group within exon 2 of MICB. The patients without the S1 group had a significantly lower risk of relapse than patients with S1 in univariate analysis (*p* < 0.01) (Figure 5), but this observation was not confirmed by multivariate analysis (*p* = 0.069 with HR = 3.764, 95% CI, 0.902–15.707).

The polymorphism which distinguishes the group S1 from the others is an exchange of lysine to glutamate at position 58 of MICB (the position is calculated without the leading sequence) (Table 7).

#### 3.3.4. Role of Known Polymorphisms Was Not Evident in Our Cohort

**Figure 6:**
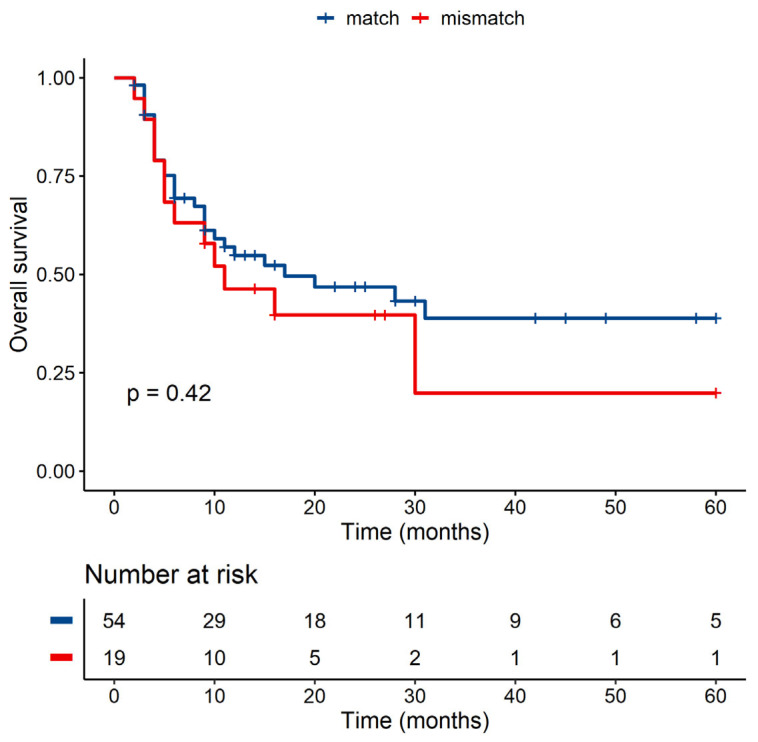
OS of patients with matched graft versus patients with mismatched graft within MICA-129.

We also analyzed the effect of known ligand polymorphisms called MICA-129 and MICB-98, and polymorphisms within the receptor NKG2D (HNK1 and LNK1). Our results did not show any difference between grafts from MICA-129Val/Val, MICA-129Val/Met or from MICA-129Met/Met donors (*p* = 0.73, data not shown), and we did not detect any difference in OS of patients with any MICA-129 combination (*p* = 0.97, data not shown). Focusing on match/mismatch, our cohort did not show any statistically significant result (*p* = 0.42) (Figure 6).

Regarding the MICB-98, we did not observe any association of MICB-98 amino acid with RFS or OS. We did not investigate the role of match and mismatch between donor and patient at MICB-98 position because of the low number of mismatched patients (five patients with mismatched graft).

We did not see any difference in HSCT outcome between grafts from HNK/HNK and grafts from HNK/LNK or LNK/LNK in our cohort.

All the above-mentioned results are summarized in Table 8 and Table 9.

## 4. Discussion

Our study was focused on the detection of single nucleotide substitutions leading to the inclusion of other amino acids within NKG2D, MICA, and MICB, and exploring their potential role in the outcome of patients.

Some amino acid changes associated with an outcome, such as MICA-129 (Val/Met) or MICB-98 (Ile/Met), were already investigated. Multiple works describe the role of MICA-129 polymorphism in GVHD development and OS. Patients’ MICA-129Val/Val genotype has an increased risk of cGVHD according to Boukouaci et al. [15]. This was also observed in our study, where 43% of patients with MICA-129Val/Val had cGVHD compared to patients with Val/Met or Met/Met, only 22% of whom developed cGVHD. In addition, Isernhagen and colleagues found that patients with at least one allele with MICA-129Met have better OS, although homozygotes with MICA-129Met/Met experience aGVHD more frequently. However, aGVHD-related mortality is lower in those patients [14]. We did not confirm this association, which is in line with other works [33,34] also not proving this connection.

Besides the role of the patient’s MICA-129, the role of the donor’s MICA-129 polymorphism was described previously [34]. Patients with a graft from a donor with at least one MICA-129Met allele should have a lower risk of NRM [34]. This trend was not seen in our study, but we observed a potential role of different polymorphism—MICA-14 (rs1063630) on OS. Patients receiving a graft from a donor who has at least one copy of MICA-14Gly (group S4/Sx) had significantly worse OS. MICA-14 polymorphism is found in exon 2 which encodes binding domain α1, which directly interacts with NKG2D. This amino acid exchange can lead to a different affinity to NKG2D, causing various reactivity of NK cells, as Chen et al. indicated in their work [35]. Because the described polymorphism played the role when presented on donors’ leukocytes, we can speculate about the higher sensitivity of activated immune cells expressing NKG2D ligands to NK cell killing [3]. 

Apart from studying MICA, the role of MICB-98 match and mismatch between patient and donor was investigated too. Unfortunately, we cannot provide any confirmation of the results from Carapito et al. [18], who stated that the MICB-98 match reduces GVHD incidence as well as an effect of CMV on HSCT outcome, because, in our set of patients/donors, we have only five mismatches, preventing statistical analysis. However, we observed the effect of an amino acid exchange (MICB-58Lys → Glu) within exon 2 of MICB. In contrast to MICA-14, where the effect depended on the donor’s polymorphism, this effect is seen in patients. Patients with at least one allele with MICB-58Glu have a significantly higher risk of relapse than patients without this allele, confirmed by univariate analysis but not by multivariate analysis. The reason why the result of multivariate analysis is not statistically significant can lie in higher representation of patients transplanted with active disease in the S1 group (51% of patients carrying S1 group vs. 19% of patients without S1). The exchange appears in a binding domain α1 of MICB, and we can speculate that this exchange can influence binding domain structure and change the affinity to the NKG2D receptor. In the case of MICA-129, patients with Met/Met had a higher risk of relapse, and MICA-129Met triggers a stronger but shorter (due to a sooner NKG2D exhaustion) reaction of NK cells than MICA-129Val [15]. Using this logic on MICB-58, we can expect that MICB-58Glu binds NKG2D similarly, i.e., with higher affinity, activating a stronger but shorter NK cell reaction, which can lead to anticancer immune reaction failure. This hypothesis can explain the decreased ability to achieve complete remission in patients carrying MICB-58Glu observed in our cohort.

The polymorphisms in MICA/MICB genes could also be associated with different cell surface expression and soluble form levels [36,37] and could potentially influence the ligand regulation on transcription [38], translation [39], or post-translation [40] level; therefore, the determination of serum level, as well as surface protein level, should be further investigated.

The last investigated polymorphisms were the HNK haplotypes of the NKG2D receptor. Espinoza et al. observed that patients with the so-called standard-risk disease have improved OS in the case that they received graft with HNK1 [11]. We did not find any statistically significant difference in HSCT outcome between grafts with or without HNK1 (*p* = 0.9). Similar results as ours can be found in the work from Apithy et al. [41].

## 5. Conclusions

We found a new polymorphism (MICA-14Gly) influencing HSCT outcome in our cohort of patients undergoing HSCT for AML. We did not observe the role of already known polymorphisms. Our results, together with the different results for already known polymorphisms, indicate the necessity of integrated multicenter studies that can better evaluate the effect of individual polymorphisms and their potential use in donor selection and risk assessment of post-transplant complications.

## Figures and Tables

**Table 1:** Clinical parameters of our cohort. * DRI was defined according to Armand et al. [20]. ** Secondary malignancy is defined as AML after any previous malignancy. *** Complex karyotype is a karyotype with ≥3 abnormalities. **** PTCY is post-transplantation cyclophosphamide. ***** HCT-CI is hematopoietic cell transplantation comorbidity index [21].

Patient Age Group		Type of Donor	
<50	32	Related donor (full match)	25
50–65	65	Unrelated donor	64
>65	27	Haploidentical donor	35
Median (years)	58	**Conditioning therapy**	
Range (years)	23–74	Myeloablative	23
**Patient’s sex**		Reduced	101
Male	72	**GVHD prophylaxis**	
Female	52	With PTCY ****	34
**Diagnosis**		Without PTCY	90
AML	118	**aGVHD**	
MDS	6	Yes	96
Disease Risk Index (DRI) *		No	28
Low	5	**cGVHD**	
Intermediate	67	Yes	38
High	33	No	64
Very high	7	Unknown	22
Unknown	12	**Relapse**	
AML as secondary malignancy **		Yes	41
Yes	37	No	82
No	87	Unknown	1
**Karyotype**		**Outcome**	
Normal karyotype	58	Dead	62
Complex karyotype ***	16	Alive	62
Other karyotype changes	11	**Cause of death**	
Unknown	39	Relapse	29
**Disease status during HSCT**		Infection	16
Active disease	43	Organ failure	7
Complete remission	81	GVHD	6
**Graft source**		Graft rejection	1
Bone marrow	24	Unknown	3
Peripheral blood stem cells	100	**HLA mismatch**	
**CMV match/mismatch**		None	73
Match	75	Haploidentical	35
Mismatch	49	ABC/DP-DR	16
**Pretransplant T-cell depletion (patient)**		**Sex match/mismatch**	
Yes	64	Match	65
No	60	Mismatch	59
**EBMT risk score**		HCT-CI *****	
1–2	24	≥3	34
3	39	<3	81
4	30	unknown	9
5–6	29		
NA	2		

**Table 2:** Information about PCR primers used in our study.

Sequence of	Length of the Sequence	PCR Primers	Published in
NKG2D-Hb1	253 bp	F: TGCGAGGTATTTATGTTCTG R: ACAGTTTAGGAATACAGCAC	[22]
NKG2D-Hb2	230 bp	F: TTAAGGCTGGAGAATAATGC	[23]
		R: TCAGTGAAGGAAGAGAAGG	
MICA	1.9 kbp (exons 2–4)	F: CCCCCTTCTTCTGTTCATCA R: TGACTCTGAAGCACCAGCAC	[24]
MICB	2.1 kbp (exons 2–5)	F: GGACAGCAGACCTGTGTGTTA R: AAAGGAGCTTTCCCATCTCC	[24]

**Table 3:** Parameters tested by Kaplan–Meier method for OS and RFS.

OS and RFS Compared within Parameters
MICA/B exons 2–4
MICA-129
MICB-98
MICA/B hetero vs. homozygosity
NKG2D haploblocks 1–2
Match and mismatch within exons 2–4 (MICA/B)
Match and mismatch within MICA-129
Match and mismatch within MICB-98
Match and mismatch NKG2D haploblocks 1–2
Each specific group of each exon compared to the other groups

**Table 4:** Results of univariate analysis; statistically significant results are bold. * HR is hazard ratio. ** CI is confidence interval.

	OS				RFS			
	HR *	Lower CI **	Upper CI	*p*-Value	HR	Lower CI	Upper CI	*p*-Value
**Patient age group**								
<50	1				1			
50–65	1.706	0.922	3.155	0.089	1.274	0.602	2.699	0.527
>65	1.105	0.490	2.494	0.809	0.552	0.173	1.763	0.316
**Patient’s sex**								
Female	1				1			
Male	0.939	0.567	1.557	0.809	0.956	0.493	1.856	0.895
**Diagnosis**								
AML	1				1			
MDS	0.332	0.046	2.398	0.275	0.563	0.077	4.109	0.571
**DRI**								
Low	1				1			
Intermediate	0.920	0.278	3.050	0.892	1.181	0.152	9.149	0.874
High	3.821	1.151	12.686	**0.029**	7.450	0.989	56.129	0.051
Very high	8.256	2.095	32.545	**0.003**	23.261	2.747	196.974	**0.004**
**AML as secondary malignancy**								
No	1				1			
Yes	2.106	1.263	3.513	**0.004**	2.188	1.123	4.261	**0.021**
**Karyotype**								
Normal karyotype	1				1			
Other changes	1.542	0.866	2.745	0.141	1.703	0.746	3.887	0.206
Complex karyotype	4.162	2.100	8.251	**<0.001**	8.404	3.658	19.306	**<0.001**
**Disease status during HSCT**								
Active disease	1				1			
Complete remission	0.527	0.320	0.869	**0.012**	0.510	0.265	0.983	**0.044**
**Graft source**								
Bone marrow	1				1			
PBSC	0.829	0.457	1.505	0.538	0.646	0.312	1.341	0.241
**Type of donor**								
Haploidentical	1				1			
Related	0.648	0.323	1.298	0.221	1.346	0.529	3.424	0.533
Unrelated	0.595	0.333	1.063	0.080	0.974	0.423	2.243	0.950
**Conditioning**								
Myeloablative	1				1			
Reduced	1.564	0.743	3.291	0.239	0.969	0.424	2.215	0.940
**GVHD prophylaxis**								
No	1				1			
Yes	1.578	0.910	2.736	0.104	0.796	0.348	1.821	0.589
**aGVHD**								
No	1				1			
Yes	1.139	0.617	2.101	0.678	0.904	0.425	1.923	0.793
**cGVHD**								
No	1				1			
Yes	0.476	0.243	0.933	**0.031**	0.232	0.080	0.673	**0.007**
**HCT-CI**								
≥3	1				1			
<3	0.753	0.436	1.302	0.311	0.850	0.414	1.745	0.659

**Table 5:** The percentage rate of survivors and dead patients, with causes of deaths for patients transplanted with or without grafts with MICA exon 2 S4 group.

	S4+ Grafts	Non-S4 Grafts
Alive patients	41%	56%
Dead patients	59%	44%
		
**Causes of death**		
Relapse	41%	54%
Infection	36%	22%
Organ failure	14%	11%
GVHD	9%	11%
Graft rejection	0%	3%

**Table 6:** Comparison of amino acid sequences of MICA exon 2 (selected part of the sequence, positions are calculated without the leading sequence). The polymorphism MICA-14Gly is labeled.

MICA Exon 2 Amino Acid Sequence from Position 3 to 37 of MICA Protein	
Group	Sequence
Group S1	HSLRYNLTVLSWDGSVQSGFLAEVHLDGQPFLRYD
Group S2	HSLRYNLTVLSWDGSVQSGFLAEVHLDGQPFLRCD
Group S4	HSLRYNLTVLS**G**DGSVQSGFLAEVHLDGQPFLRCD
Group S6	HSLRYNLTVLSWDGSVQSGFLTEVHLDGQPFLRCD
Group S7	HSLPYNLTVLSWDGSVQSGFLAEVHLDGQPFLRYD

**Table 7:** Comparison of the amino acid sequences of MICB exon 2 (selected part of the sequence, positions are calculated without the leading sequence). The polymorphism MICB-58Glu is labeled.

MICB Exon 2 Amino Acid Sequence from Position 49 to 63 of MICB Protein	
Group	Sequence
Group S1	QWAEDVLGA**E**TWDTE
Group S2	QWAEDVLGAKTWDTE
Group S3	QWAENVLGAKTWDTE

**Table 8:** Results of multivariate analysis for OS; statistically significant results are bold.

MICA Exon 2—Comparison of Group Combinations				
	*p*-Value	Hazard Ratio	Lower CI*	Upper CI
S1/S1		1		
S1/S2	2.062	0.427	0.088	2.062
S1/S4	0.028	2.745	1.113	6.771
S1/S6	0.120	0.193	0.024	1.538
**MICA exon 2 (MICA-14Gly)—MICA-14Gly (S4) presence versus absence (non-S4)**				
	***p*-Value**	**Hazard ratio**	**Lower CI**	**Upper CI**
Non-S4		1		
S4+	0.035	2.254	1.058	4.801
**MICA-129 match/mismatch**				
	***p*-Value**	**Hazard ratio**	**Lower CI**	**Upper CI**
Match		1		
Mismatch	0.155	2.407	0.718	8.069

**Table 9:** Results of multivariate analysis for RFS.

Donor—MICB Homozygote Versus Heterozygote				
	*p*-Value	Hazard Ratio	Lower CI*	Upper CI
Homozygote		1		
Heterozygote	0.433	1.693	0.454	6.309
**Patient—MICB Homozygote Versus Heterozygote**				
	***p*-Value**	**Hazard ratio**	**Lower CI**	**Upper CI**
Homozygote		1		
Heterozygote	0.322	2.101	0.483	9.134
**MICB Exon 3—Heterozygotes (S1/S3 + S2/S3) Versus Homozygote (S3/S3)**				
	***p*-Value**	**Hazard ratio**	**Lower CI**	**Upper CI**
S1/S3 + S2/S3		1		
S3/S3	0.376	0.506	0.112	2.286
**MICB Exon 2 (MICB-58Glu)—MICB-58Glu (S1) Presence Versus Absence (non-S1)**				
	***p*-Value**	**Hazard ratio**	**Lower CI**	**Upper CI**
Non-S1		1		
S1+	0.069	3.764	0.902	15.707

## Data Availability

With respect to the internal policy of the transplant center and local personal data protection rules, all data are available on request. All data will be provided upon official aaplication. Please contact holubovam@fnplzen.cz.

## Supplementary Materials

The following are available online at https://www.mdpi.com/article/10.3390/jcm10204636/s1, Tables S1–S6: Amino acid sequences of MICA and MICB distributed into the groups.
