# Supplementary material for: Single-Nucleotide Polymorphisms in MICA and MICB Genes Could Play a Role in the Outcome in AML Patients after HSCT
Source: J Clin Med. 2021 Oct 9;10(20):4636. doi: 10.3390/jcm10204636 (PMC8537017; doi:10.3390/jcm10204636)
Supplement: Supplementary file 1 [file jcm-10-04636-s001.zip › jcm-1353434-supplementary.pdf]

MICA

**Group S1**	**PHSLRYNLTVLSWDGSVQSGFLAEVHLDGQPFLRYDRQKCRAKPQGQW<br>AEDVLGNKTWDRETRDLTGNGKDLRMTLAHIKDQKE**
**Group S2**	**PHSLRYNLTVLSWDGSVQSGFLAEVHLDGQPFLRCDRQKCRAKPQGQW<br>AEDVLGNKTWDRETRDLTGNGKDLRMTLAHIKDQKE**
Group S3	PHSLRYNLTVLSGDGSVQSGFLAEGHLDGQPFLRCDRQKCRAKPQGQWAED<br>VLGNKTWDRETRDLTGNGKDLRMTLAHIKDQKE
**Group S4**	**PHSLRYNLTVLSGDGSVQSGFLAEVHLDGQPFLRCDRQKCRAKPQGQW<br>AEDVLGNKTWDRETRDLTGNGKDLRMTLAHIKDQKE**
Group S5	PHSLRYNLTVLSWDGSVQSGFLAEGHLDGQPFLRCDRQKCRAKPQGQWAE<br>DVLGNKTWDRETRDLTGNGKDLRMTLAHIKDQKE
**Group S6**	**PHSLRYNLTVLSWDGSVQSGFLTEVHLDGQPFLRCDRQKCRAKPQGQW<br>AEDVLGNKTWDRETRDLTGNGKDLRMTLAHIKDQKE**
**Group S7**	**PHSLPYNLTVLSWDGSVQSGFLAEVHLDGQPFLRYDRQKCRAKPQGQW<br>AEDVLGNKTWDRETRDLTGNGKDLRMTLAHIKDQKE**
Group S8	PQSSL*PHGAVLGWICAVRVSC*GTSGWSALPAL*QAEMQGKAPGTVGRRCP<br>GK*DMGQRDQGLDRERKGPQDDPGSYQGPERR
Group S9	PHSLRYNLTVLSGDGSVQSGFLAEVHLDGQPFLRCDRQKGRAKPQGQWAED<br>VLGNKTWDRETRDLTGNGKDLRMTLAHIKDQKE
Group S10	PHSLRYNLTVLSGDGSVQSGFLAEASGWSALPAL*QAEMQGKAPGTVGRRCP<br>GK*DMGQRDQGLDRERKGPQDDPGSYQGPERR

Table S1. Amino acid sequences of MICA exon 2 groups. Groups found in our cohort are bold.

**Group S1**	**LHSLQEIRVCEIHEDNSTRSSQHFYYDGELFLSQNLETEEWTVPQSSRAQ<br>TLAMNVRNFLKEDAMKTKTHYHAMHADCLQELRRYLESGVVLRRT**
**Group S2**	**LHSLQEIRVCEIHEDNSTRSSQHFYYDGELFLSQNLETEEWTMPQSSRA<br>QTLAMNIRNFLKEDAMKTKTHYHAMHADCLQELRRYLKSGVVLRRT**
Group S3	LHSLQEIRVCEIHEDNSTRSSQHFYYDGELFLSQNLESEEWTVPQSSRAQTL<br>AMNVRNFLKEDAMKTKTHYHAMHADCLQELRRYLESSVVLRRT
Group S4	LHSLQEIRVCEIHEDNSTRSSQHFYYDGELFLSQNLETEEWTMPQSSRAQTL<br>AMNVRNFLKEDAMKTKTRYHAMHADCLQELRRYLKSGVVLRRT
**Group S5**	**LHSLQEIRVCEIHEDNSTRSSQHFYYDGELFLSQNLETEEWTMPQSSRA<br>QTLAMNVRNFLKEDAMKTKTHYHAMHADCLQELRRYLKSGVVLRRT**
**Group S6**	**LHSLQEIRVCEIHEDNSTRSSQHFYYDGELFLSQNVETEEWTVPQSSRA<br>QTLAMNVRNFLKEDAMKTKTHYHAMHADCLQELRRYLESSVVLRRT**
**Group S7**	**LHSLQEIRVCEIHEDNSTRSSQHFYYDGELFLSQNVETEEWTVPQSSRA<br>QTLAMNVRNFLKEDAMKTKTHYHAMHADCLQELRRYLESSVVLRRR**
**Group S8**	**LHSLQEIRVCEIHEDNSTRSSQHFYYDGELFLSQNLETEEWTVPQSSRAQ<br>TLAMNVRNFLKEDAMKTKTHYHAMHADCLQELRRYLESSVVLRRT**
**Group S9**	**LHSLQEIRVCEIHEDNSTRSSQHFYYDGELFLSQNLETEEWTMPQSSRA<br>QTLAMNVRNFLKEDAMKTKTLYHAMHADCLQELRRYLKSGVVLRRT**

Group S10	LHSLQEIRVCEIHEDNSTRSSQHFYYDGELFLSQNLETEEWTMPQSSRAQTL AMNVRNFLKEDAMKTKTHYHAMHADCLQELRRYLKSSVVLRRT
Group S11	LHSLQEMRVCEIHEDNSTRSSQHFYYDGELFLSQNVETEEWTVPQSSRAQT LAMNVRNFLKEDAMKTKTHYHAMHADCLQELRRYLESSVVLRRT
Group S12	LHSLQEIRVCEIHEDNSTRSSQHFYYDGELFLSQNVETEEWTVPQSSRAQTL AMNVRNFLKEDAMKTKTHYHAMHADCLQELRRYLESSIVLRRT
**Group S13**	**LHSLREIRVCEIHEDNSTRSSQHFYYDGELFLSQNLETEEWTMPQSSRA QTLAMNVRNFLKEDAMKTKTHYHAMHADCLQELRRYLKSGVVLRRT**
Group S14	LHSLQEIRVCEIHEDNSTRSSQHFYYDGELFLSQNVETEEWTVPQSSRAQTL AMNVRNFLKEDAMKTKTHYHAMHADCLQELRRYLESSVVLRT-
**Group S15**	**LHSLQEIRVCEIHEDNSTRSSQHFYYDGELFLSQNLETKEWTMPQSSRA QTLAMNVRNFLKEDAMKTKTHYHAMHADCLQELRRYLKSGVVLRRT**
**Group S16**	**LHSLQEIRVCEIHEDNSTRSSQHFYYDGELFLSQNLETEEWTMPQSSRA QTLAMNVRNFLKEDAVKTKTHYHAMHADCLQELRRYLKSGVVLRRT**
Group S17	LHSLQEIRVCEIHEDNSTRSSQHFYYDGELFLSQNVETEEWTVPQSSRAQTL AMNVRNFLKEDAMKTKTHYHAMHADCLQELRRYLESGVVLRRR
Group S18	LHSLQEIRVCEIHEDNSTRSSQHFYYDRELFLSQNLETEEWTMPQSSRAQTL AMNVRNFLKEDAMKTKTHYHAMHADCLQELRRYLKSGVVLRRT

Table S2. Amino acid sequences of MICA exon 3 groups. Groups found in our cohort are bold.

**Group S1**	**PPMVNVTRSEASEGNITVTCRASGFYPWNITLSWRQDGVSLSHDTQQW GDVLPDGNGTYQTWVATRICQGEEQRFTCYMEHSGNHSTHPVPS**
**Group S2**	**PPMVNVTRSEASEGNITVTCRASSFYPRNITLTWRQDGVSLSHDTQQW GDVLPDGNGTYQTWVATRICQGEEQRFTCYMEHSGNHSTHPVPS**
**Group S3**	**PPMVNVTRSEASEGNITVTCRASSFYPRNIILTWRQDGVSLSHDTQQWG DVLPDGNGTYQTWVATRICRGEEQRFTCYMEHSGNHSTHPVPS**
Group S4	PPMVNVTRSEASEGNITVTCRASGFYPWNITLSWRQDGVSLSHDTQQWGD VLPDGNGTYQTWVATRICQGEEQSFTCYMEHSGNHSTHPVPS
**Group S5**	**PPMVNVTRSEASEGNITVTCRASGFYPWNITLSWRQDGVSLSHDTQQW GDVLPDGNGTYQTWVATRICEGEEQRFTCYMEHSGNHSTHPVPS**
**Group S6**	**PPMVNVTRSEASEGNITVTCRASGFYPWNITLSWRQDGVSLSHDTQQW GDVLPDGNGTYQTWVATRICQGEEQRFTCYMEHSGNHSTHAVPS**
Group S7	PPMVNVTRSEASEGNITVTCRASSFYPRNIILTWRQDGVSLSHDTQQWGDV LPDGNGTYQTWVATRICQGEEQRFTCYMEHSGNHSTHPVPS
Group S8	PPMVNVTRSEASEGNITVTCRASGFYPWNITLSWRQDGVSLSHDTQQWGD VLPDGNGTYQTWVATRICQGEEQRFTCYMEHSRNHSTHPVPS
Group S9	PPMVNVTRSEASEGNITVTCRASSFYPRNIILTWRQDGVSLSHDTQQWGDV LPDGNGTYQTWVATRICRGEEQRFTCYMEHSRNHSTHPVPS
**Group S10**	**PPMVNVTRSEASEGNITVTCRASSFYPRNIILTWRQDGLSLSHDTQQWG DVLPDGNGTYQTWVATRICRGEEQRFTCYMEHSGNHSTHPVPS**

Table S3. Amino acid sequences of MICA exon 4 groups. Groups found in our cohort are bold.

MICB

**Group S1**	**PHSLRYNLMVLSQDGSVQSGFLAEGHLDGQPFLRYDRQKRRAKPQGQWAEDVLGAETWDTETEDLTENGQDLRRTLTHIKDQKG**
**Group S2**	**PHSLRYNLMVLSQDGSVQSGFLAEGHLDGQPFLRYDRQKRRAKPQGQWAEDVLGAKTWDTETEDLTENGQDLRRTLTHIKDQKG**
**Group S3**	**PHSLRYNLMVLSQDGSVQSGFLAEGHLDGQPFLRYDRQKRRAKPQGQWAENVLGAKTWDTETEDLTENGQDLRRTLTHIKDQKG**
Group S4	PHSLRYNLMVLSQDGSVQSGFLAEGHLDGQPFLRYDRQKRRAKPRDSGQKMSWELRPGTQRPRT*QRMGKTSGGP*LISRTRKE
**Group S5**	**PHSLRYNLMVLSQDGSVQSGFLAEGHLDGQSFLRYDRQKRRAKPQGQWAENVLGAKTWDTETEDLTENGQDLRRTLTHIKDQKG**
Group S6	PHSLRYNLMVLSQDGSVQSGFLAEGHLDGQPFLRYDRQKCRAKPQGQWAEDVLGAETWDTETEDLTENGQDLRRTLTHIKDQKG

Table S4. Amino acid sequences of MICB exon 2 groups. Groups found in our cohort are bold.

**Group S1**	**LHSLQEIRVCEMHEDSSTRGSRHFYYNGELFLSQNLETQESTVPQSSRAQTLAMNVTNFWKEDAMKTKTHYRAMQADCLQKLQRYLKSGVAIRRT**
**Group S2**	**LHSLQEIRVCEIHEDSSTRGSRHFYYNGELFLSQNLETQESTVPQSSRAQTLAMNVTNFWKEDAMKTKTHYRAMQADCLQKLQRYLKSGVAIRRT**
**Group S3**	**LHSLQEIRVCEIHEDSSTRGSRHFYYDGELFLSQNLETQESTVPQSSRAQTLAMNVTNFWKEDAMKTKTHYRAMQADCLQKLQRYLKSGVAIRRT**
Group S4	LHSLQEIRVCEIHEDSSTRGSRHFYYDGELFLSQNLETQESTVPQSSRAQTLAMNVTNFWKEDAMKTKTHYRAMQADCLQKLQRYLKSGGPSGEQ
Group S5	LHSLQEIRVCEIHEDSSTRGSRHFYYDGELFLSQNLETQESTVPQSSRAQTLAMNVTNFWKEDAMKTKTHYRAMQADCLQKLQ*YLKSGVAIRRT
Group S6	LHSLQEIRVCEIHEDSSTRGSRHFYYDGELFLSQNLETQESTVPQSSRAQTLAMNVTNFWEEDAMKTKTHYRAMQADCLQKLQRYLKSGVAIRRT

Table S5. Amino acid sequences of MICB exon 3 groups. Groups found in our cohort are bold.

**Group S1**	**PPMVNVICSEVSEGNITVTCRASSFYPRNITLTWRQDGVSLSHNTQQWGDVLPDGNGTYQTWVATRIRQGEEQRFTCYMEHSGNHGTHPVPS**
**Group S2**	**PPMVNVTCSEVSEGNITVTCRASSFYPRNITLTWRQDGVSLSHNTQQWGDVLPDGNGTYQTWVATRIRQGEEQRFTCYMEHSGNHGTHPVPS**
**Group S3**	**PPMVNVTCSEVSEGNITVTCRASSFYPRNITLTWRQDGVSLSHNTQQWGDVLPDGNGTYQTWVATRIRQGEEQRFTCYMEHSGNHSTHPVPS**

Table S6. Amino acid sequences of MICB exon 4 groups. Groups found in our cohort are bold.
